# Breast cancer primary tumor ER expression pattern predicts its expression concordance in matched synchronous lymph node metastases

**DOI:** 10.1186/s12885-018-5217-5

**Published:** 2018-12-27

**Authors:** Juan Zhao, Chunxiu Hu, Cheng Wang, Wei Yu, Yinglu Guo, Minghan Shi, Yongjie Shui, Qichun Wei

**Affiliations:** 1 Department of Radiation Oncology, the Second Affiliated Hospital, Zhejiang University School of Medicine, Jiefang Road 88, Hangzhou, 310009 People’s Republic of China; 2 Department of Pathology, Shaoxing People’s Hospital (Shaoxing Hospital, Zhejiang University School of Medicine), Shaoxing, 312000 People’s Republic of China; 3 Ministry of Education Key Laboratory of Cancer Prevention and Intervention, Zhejiang University School of Medicine, Hangzhou, 310009 People’s Republic of China

**Keywords:** Breast cancer, Metastasis, Estrogen receptor, Concordance

## Abstract

**Background:**

Estrogen receptor (ER) expression is important for treatment selection and prognostication of breast cancer patients. Although the metastases are the main targets of endocrine therapy, ER status is often based on the primary tumor. However, ER expression in breast cancer primary lesion may not match with its synchronous metastatic lesions in some cases. In this study, we analyzed ER expression concordance between breast cancer primary tumor and metastatic lesions.

**Methods:**

Paraffin blocks of 100 primary breast invasive ductal carcinoma cases with axillary lymph node metastases were collected. Five tissue cores were punched out from individual primary breast cancer, and one tissue core from each lymph node metastases to assemble tissue microarrays for ER staining. Samples were then scored as 0, 1+, 2+, and 3+ according to the number and intensity of ER stained tumor cells.

**Results:**

For cases with ER 3+ (strong expression) in all cores of primary lesions (*n* = 38), ER expression in metastatic lymph node was found in 94.7% of the patients. 91.0% of the metastatic lymph nodes were ER positive, and 84.3% of them to be 3+. Among the 46 cases of ER negative expression in all cores of primary lesions, 39 of them had all the metastatic nodes being ER negative, and ER negative nodes were seen in 95.7% of the metastases. As for 16 cases of ER inconsistent expression in primary lesions, 4 cases showed negative ER expression in all metastatic nodes, 2 cases displayed diffuse consistent ER 3+ expression, and 10 cases displayed variant ER expression.

**Conclusions:**

The findings suggest that ER expression concordance between breast cancer primary lesion and its matched metastatic lesions could be estimated by primary tumor ER expression pattern.

## Background

Estrogen receptor (ER) expression is important for treatment selection and prognostication of breast cancer patients. Although the metastases are the main targets of endocrine therapy, ER status is often based on the primary tumor. However, ER expression in breast cancer primary lesion may not match with its synchronous metastatic lesions in some cases. There are several studies reported the changes of ER expression in the metastatic tumors when compared to the primaries [1–5]. In some studies, change of ER status was regarded to be rare for metastasis [2, 4], whereas others found that metastatic tumors are different from the primary ones in ER expression [1, 3, 5].

Biological explanations for discrepant ER expression in metastases have been mentioned, including heterogeneity of the primary lesion, cancer cells early distant seeding, clonal selection, and therapy induced clonal evolution [6, 7]. Whether the extensity and intensity of ER positive cells in the primary lesion influence the ER status of the metastases, so far, has not been investigated.

In this study, multiple tissue cores were punched from each of 100 breast invasive ductal carcinoma primary lesion. Tissue core was also punched from every individual metastatic lymph node. The aim of the study was to analyze the agreement of ER expression among different tissue cores from the same primary lesion and each individual metastatic lymph node. The effect of ER positive cells extensity on ER expression agreement was also studied.

## Methods

### Patients and samples

This study was conducted with the approval of the Shaoxing Hospital Institutional Review Board. Informed consent was obtained from all the patients before surgery regarding the data and samples to be used for research. All study procedures were carried out in accordance with the ethical standards of the Helsinki Declaration. Breast invasive ductal carcinoma patients treated with mastectomy or lumpectomy, and standard level I/II axillary lymph node dissection were reviewed. Archival specimens from primary tumor and lymph node metastases were reviewed independently by two pathologists to confirm the histological diagnosis and tumor grade. Patients with both primary tumor and lymph node metastases samples were included, those received any neoadjuvant therapy were excluded. One hundred qualified patients were finally identified in the period from Jan. 2008 to Oct. 2014. Forty patients were under the age of 50 years old. The cases with high, moderate, and low differentiation were 1, 53, and 46 cases, respectively. Those with T1, T2, and T3 disease were 28, 65, and 7 cases. Sixty patients had 4 or more metastatic lymph node.

### Tissue microarray (TMA) construction

A manual tissue microarrayer (TM-1, Beijing Boyikang Laboratory Instrument Limited Company, Beijing, China) was used to construct the TMAs. Five 2-mm-diameter tissue cores were punched out from the representative areas of invasive carcinoma of the donor blocks. For lymph node metastases, 2-mm-diameter tissue core was punched out from the representative area of each metastatic node. The cores were transferred into the pre-punched hole in the recipient block according to the location on the TMA map. From each TMA block, 4-μm sections were cut on a microtome (Leica RM2245, German Leica Instruments Limited Company, German) and transferred to adhesive-coated slides. One section from each tissue array block was stained with hematoxylin and eosin, and core loss or gain assessed.

### ER staining and scoring

The sections were deparaffinized in xylene and hydrated through graded concentrations of ethanol to distilled water. Following the antigen retrieval, slides were incubated in 3% H_2_O_2_ for 10 min. After being washed, the slides were incubated overnight with primary antibody (diluted 1:400) directed against estrogen receptor alpha (clone SP1, Maixin biotechnology co., LTD, Fuzhou, China) at 4 °C. After the sections were incubated in secondary antibody, the slides were finally counterstained with hematoxylin and mounted. As positive controls we used in house positive control tissue sections as well as commercially supplied positive control sections. Known ER-positive breast cancer tissue cores were used as well. As negative controls, PBS was used instead of the primary antibody. Controls were included in each staining batch.

The ER-score was graded according to the percentage of nuclei stained tumor cells and the intensity of the staining. The proportion of positive cells was scored using a scale of 0–4, where 0 corresponded to no tumor cells were stained, 1 corresponded to < 10%, 2 corresponded to 11–50%, 3 corresponded to 51–80%, 4 corresponded to > 80% of the tumor cells were stained. The staining intensity was scored as 0, 1, 2 or 3. The TMA was evaluated independently by two pathologists (J.Z. and C.W.). In case of a discrepancy, the 2 observers simultaneously reviewed the slides under a multi-headed microscope to achieve a consensus. The immunoreactive score (IRS) is the product of a proportion score and an intensity score with a range of 0–12. The ER staining was then graded as negative expression, 0+ (IRS of 0 and 1); weak expression, 1+ (IRS of 2 and 3); moderate expression, 2+ (IRS of 4, 6 and 8); strong expression, 3+ (IRS of 9 and 12).

## Results

### ER expression of primary tumors and the concordance among different cores

A total of 498 cores were punched from 100 breast cancer primary lesion blocks, and tumor cells were identified in 472 cores. In 54 of 100 (54%) patients, immunostaining for ER was found in at least one core of the primary tumors. Accordingly, negative ER staining was seen in the rest 46 (46%) cases.

**Fig. 1:**
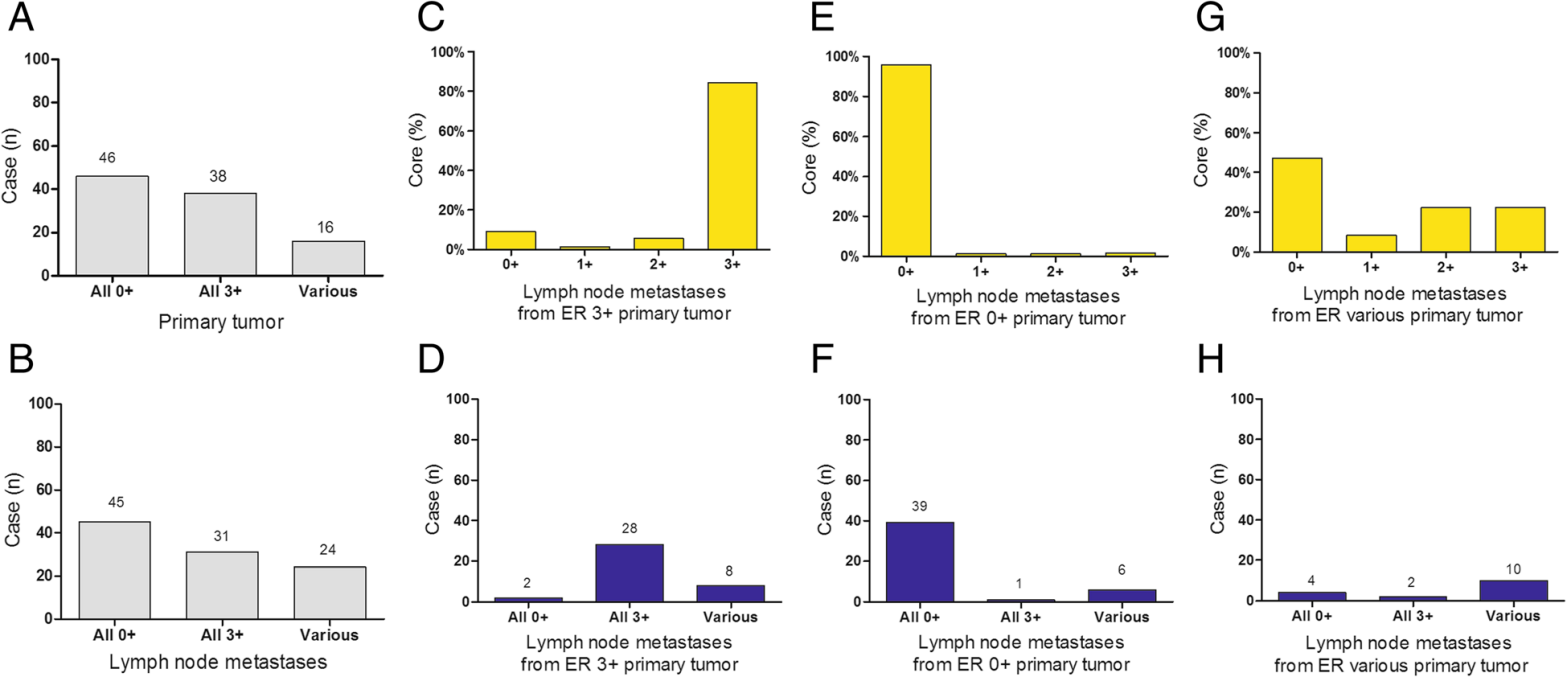
ER score in primary tumor and lymph node metastases. **a**: The cases in which all cores obtained were found to be ER negatively stained (0+), strongly expressed (3+), or various ER score in primary tumor. **b**: The cases in which all cores obtained were found to be ER negatively stained (0+), strongly expressed (3+), or various ER score in lymph node metastases. **c**, **e**, and **g**: The percentage of ER expression (0+, 1+, 2+, 3+) in lymph node metastases from patients with all cores obtained to be ER strongly expressed (**c**), negatively stained (**e**), or various ER score (**g**) in primary tumor. **d**, **f**, and **h**: ER expression status in lymph node metastases from patients with all cores obtained to be ER strongly expressed (**d**), negatively stained (**f**), or various ER score (**h**) in primary tumor

When all of the 472 tumor cell positive cores were analyzed, the patients were classified into three groups (Fig. 1a). One group included 38 cases, all cores obtained were found to be ER strongly expressed (3+). In another group of 46 primary lesions, all cores obtained were ER negatively stained. The third group is the rest 16 cases, a total of 80 cores were taken from the primary lesion blocks, tumor cells were found in 78 cores. Among the 78 tumor cell positive cores, the ER expression levels ranged as 3+, 2+, 1+ or 0 were found in 24 (30.8%), 30 (38.5%), 12 (15.4%), and 12 (15.4%) cores, respectively. The staining details of each core from all these 16 cases are shown in Table 1.

**Table 1:** Cases with inconsistent cores ER expression status in primary lesions (*n* = 16)

Case ID	N of cores (3+)	N of cores (2+)	N of cores (1+)	N of cores (0+)	N of cores No tumor
1	3	1	0	1	0
2	2	1	0	1	1
3	0	3	1	1	0
4	1	2	1	1	0
5	1	3	1	0	0
6	2	3	0	0	0
7	2	2	0	1	0
8	0	3	1	0	1
9	0	3	2	0	0
10	2	1	0	2	0
11	0	2	3	0	0
12	0	3	2	0	0
13	4	1	0	0	0
14	3	1	0	1	0
15	4	1	0	0	0
16	0	0	1	4	0
Total	24	30	12	12	2

### ER expression of lymph node metastases and the concordance among different nodes

Totally, 687 axillary lymph metastases were dissected from 100 breast cancer patients, and were punched for ER staining, tumor cells could be seen in 627 cores. In 55 of 100 (55%) analyzed patients, positive ER expression was evident in at least one metastatic node core, and for the other 45 cases, all metastatic lymph nodes were scored as ER negative.

As shown in Fig. 1b, when all the 627 metastatic lymph node cores from the 100 breast cancer patients were analyzed, 31 cases were ER strongly expressed (3+) in all the node cores, while 45 cases were ER negative stained in all the cores. For the remaining 24 cases, 212 axillary lymph metastases cores were taken. The ER expression scored as 3+, 2+, 1+ or 0 were found in 60 (28.3%), 34 (16.0%), 13 (6.1%), and 105 (49.5%) cores, respectively. The staining details of each core from all these 24 cases are shown in Table 2.

**Table 2:** Cases with inconsistant cores ER expression status in metastatic lymph nodes (*n* = 24)

Case ID	N of nodes All	N of cores (3+)	N of cores (2+)	N of cores (1+)	N of cores (0)
1	22	19	2	0	1
2	13	9	3	0	1
3	11	8	0	1	2
4	10	2	6	0	2
5	9	4	2	1	2
6	9	2	0	0	7
7	2	1	0	0	1
8	7	1	1	1	4
9	5	3	1	1	0
10	5	4	0	1	0
11	8	2	6	0	0
12	7	2	1	1	3
13	6	0	5	0	1
14	3	2	0	1	0
15	1	0	1	0	0
16	1	0	1	0	0
17	1	0	1	0	0
18	10	0	0	2	8
19	22	0	1	0	21
20	22	0	1	0	21
21	18	1	0	0	17
22	10	0	1	0	9
23	6	0	1	3	2
24	4	0	0	1	3
Total	212	60	34	13	105

### Comparison of the ER status between primary tumors and lymph node metastases

**Fig. 2:**
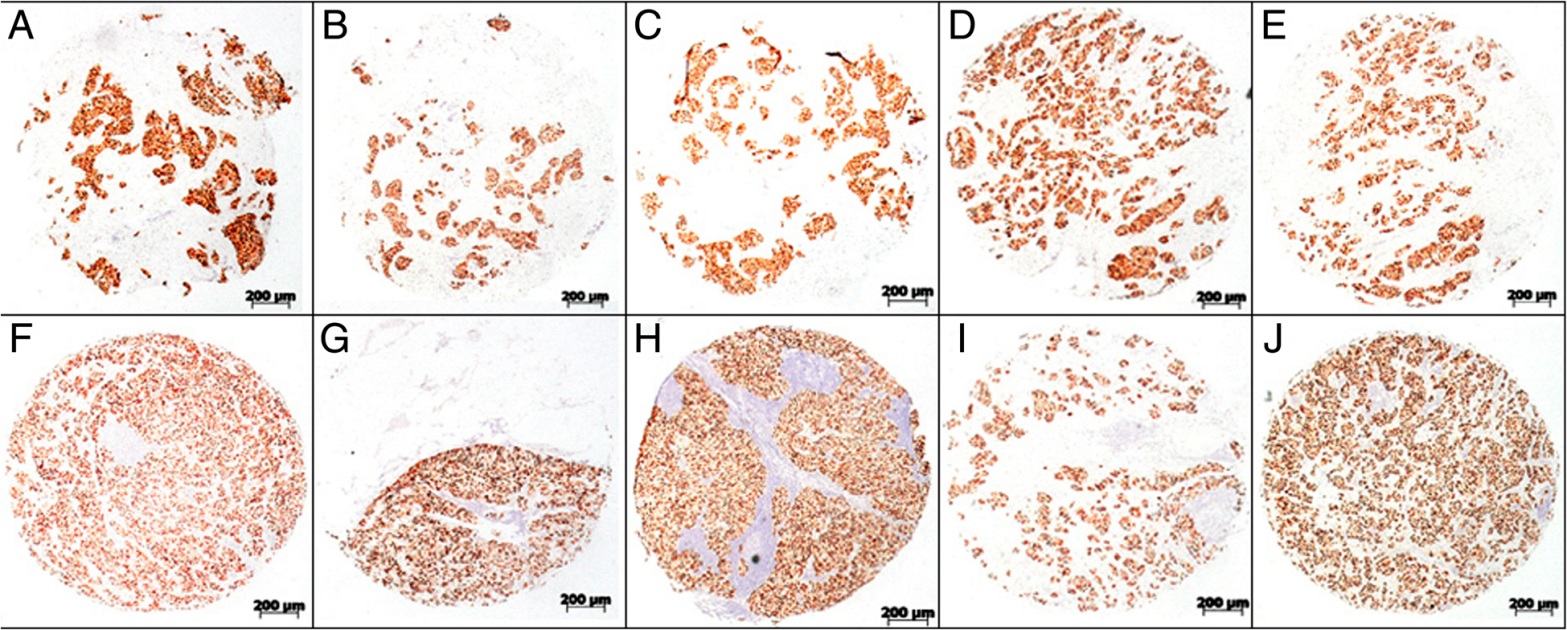
Examples of concordant immunohistochemical brown stainings of breast primary tumor and corresponding metastases. These samples were from the same patient, all the primary tumor cores (**a**, **b**, **c**, **d** and **e**) were ER-stained and scored 3+, corresponding metastatic sites (**f**, **g**, **h**, **i** and **j**) were all scored as 3+ ER expression as well. This case shows concordant 3+ ER expression among different tissue cores from the same primary lesion and every individual metastatic site

In all the primary lesions samples with ER 3+ staining, 36 out of 38 (94.7%) patients had ER positive lymph node metastases. A total of 255 cores of lymph node metastases were analyzed from these 38 patients. Among them, 215 (84.3%) cores had ER expression scored 3+, 14 (5.5%) had ER expression scored 2+, and 3 (1.2%) had ER expression scored 1+. Taken together, positive ER expression (3+, 2+ or 1+) was found in 91.0% (232/255) of the lymph node metastases. Accordingly, negative ER staining was seen in 23 (9.0%) of the lymph node metastases (Fig. 1c). As shown in Fig. 1d, in 28 out of the 38 cases, all the metastatic nodes were scored as 3+ ER expression (Fig. 2). In 2 cases, all nodes were ER negatively stained. And the rest of 8 cases had uniform 3+ expression in the primary lesions, metastatic lymph nodes with 3+ ER expression were found in all cases, however lower ER expression (2+, 1+ or 0) were also seen in the nodes. Among the 83 metastatic lymph node cores obtained from these 8 cases, ER expression scored as 3+, 2+, 1+ or 0 were respectively found in 46 (55.4%), 14 (16.9%), 3 (3.6%), and 20 (24.1%) cores.

In the 46 ER negative in all the primary lesions samples group, totally 300 lymph node metastases were taken through axillary dissection, 287 (95.7%) cores had negative ER expression, 4 (1.3%) had ER expression scored as 1+, another 4 (1.3%) had 2+, and 5 (1.7%) had ER expression scored as 3+ (Fig. 1e). As shown in Fig. 1f, 39 out of 46 cases (84.8%) were scored as ER negative expression in all the metastatic nodes. In one case, all nodes were ER 3+. In the other 6 cases, as shown in Table 2 (case 19–24), 82 metastatic lymph nodes were dissected, 73 node cores (89.0%) were found to be ER negative, the ER positive cores ranged as 1+, 2+ or 3+ were 4 (4.9%), 4 (4.9%), and 1 (1.2%) respectively.

**Fig. 3:**
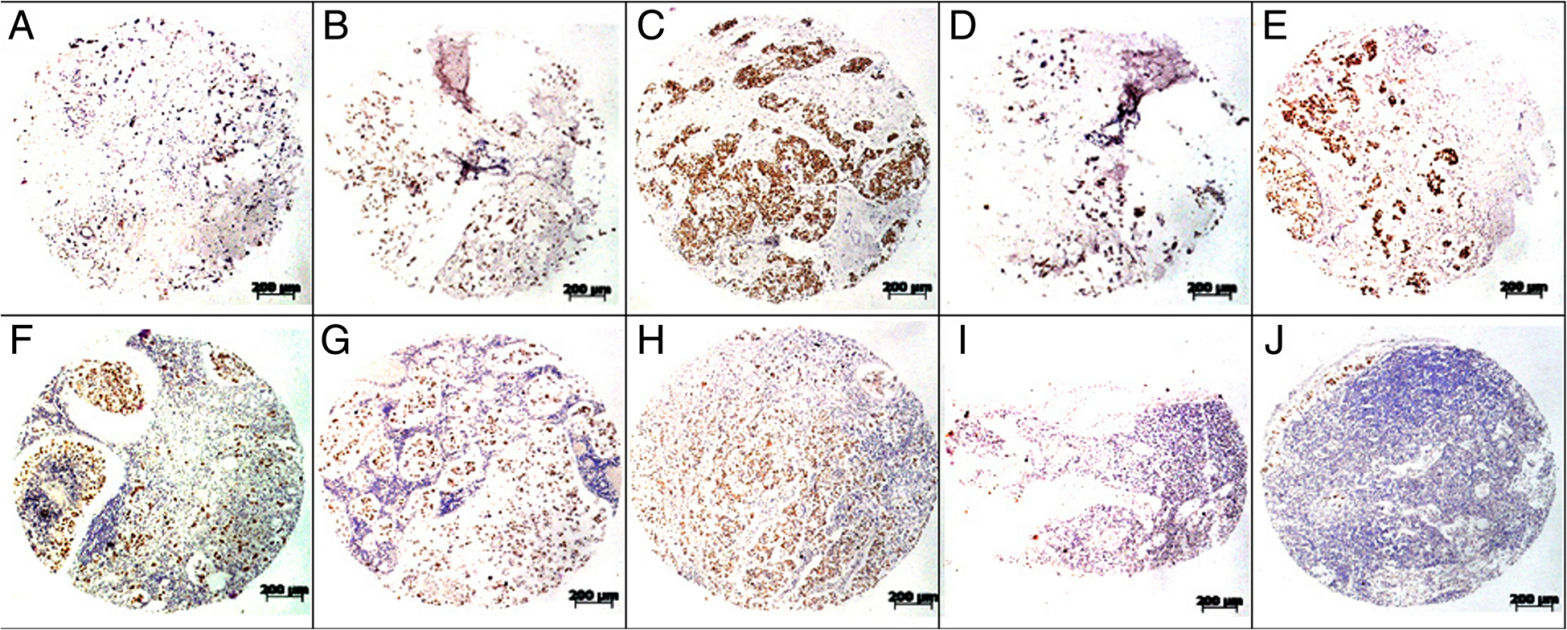
Examples of various immunohistochemical stainings of breast primary tumor and corresponding metastases. These samples were from the same patient, variant ER expression was seen in the primary tumor cores (**a**, **b**, **c**, **d** and **e**), different ER expression was also found in the corresponding metastatic sites (**f**, **g**, **h**, **i** and **j**). This case shows various ER expressions among different tissue cores from the same primary lesion and every individual metastatic lymph node

In the remaining 16 cases ER expression was disagreement among the cores from each primary lesion (as shown in Table 1). A total of 72 lymph node metastases were found through axillary dissection. As shown in Fig. 1 g and h, among the 16 cases, 4 of them were ER negative in all the detected 22 nodes, 12 cases were seen positive ER expression in the metastatic nodes. Within the 12 cases, 2 with all the nodes strongly expressed ER 3+, the other 10 cases had different ER expression levels, as shown in Table 2 (case 9–18). 47 metastatic nodes were found from the 10 cases, the nodes with ER expression level scored as 3+, 2+, 1+ or 0 were 13 (27.7%), 16 (34.0%), 6 (12.8%), and 12 (25.5%), respectively. Examples of variant ER expression in primary tumor and the corresponding metastatic sites are shown in Fig. 3.

## Discussion

In the present study, special attention was paid to the ER expression agreement among different tissue cores from the same primary lesion and their individual metastatic lymph node. The effect of ER expression pattern on ER expression concordance was studied. We found that ER expression concordance between breast cancer primary lesion and its matched synchronous lymph node metastases could be estimated by primary tumor ER expression pattern.

For patients with 3+ ER staining in all primary lesion cores, positive ER in the metastatic lymph nodes was found in about 95% of the patients. When each individual lymph node was analyzed, more than 90% of the metastatic lymph nodes were found to be ER positive in this patient group, and majority of them were 3+. For those with primary lesions being negative of ER staining in all the punched cores, although metastatic lymph node ER status gained in few cases, more than 95% of the nodes was ER negative. However, for those ER expression level varied among different punched cores from primary lesions, one quart of patients presented with ER negative lymph node metastases, one eighth with all the nodes to be strongly ER stained, about 60% with nodes expressed ER in different intensity. High concordance of ER status between the primary lesions and the paired metastatic lymph nodes could be expected in patients with uniform ER expression in all the punched cores from primary lesions, no matter it is strong ER expression or negative of ER at all. For those ER expressed in variant intensity in the primary tumor, changes of ER status in lymph node metastases were more frequent. Thus for the first time, we found that ER expression concordance between breast cancer primary lesion and its matched synchronous metastatic lesions could be estimated by primary tumor ER expression pattern.

According to our finding, diffused strong staining is the most common pattern of ER expression. Around 80% of the ER positive breast cancer cases scored as 3+ in all the punched cores. Positive ER staining in all the cores regardless of the expression extensity (3+, 2+, and 1+), was identified in up to 10% of the cases. For the other 10% of the cases, both positive and negative ER staining was found in some of the cores from the same primary lesions. In clinical practice, it is important to keep in mind of the pattern of ER expression, as it correlated with the ER status in the metastases which matters of the response to endocrine therapy in breast cancer.

The frequency of ER expression in breast cancer has been reported to vary from 66.3% up to about 80% [8–11]. Sofi et al. reported an ER expression rate of 66.3% in 132 assessed invasive ductal carcinoma patients [8]. Thompson et al. reported ER expression rates of 79.6 and 73.7% in primary lesions and metastatic lesions, respectively [9]. In the present study, ER expression rate is lower than what previously reported. This is because, to perform our study, only those cases with lymph nodes metastases were selected. Zhu et al. and Yi et al. also reported lower ER expression rate in cases with lymph nodes metastases than those without metastases [10, 11].

The general ER expression discordance is observed in 13% of the paired samples, 7.0% of the cases gained ER expression in the matched synchronous metastatic nodes, and 6.0% lost. The frequency of ER expression discrepancy between the primary lesions and the paired metastases has been reported to vary from 2.6% up to about 22.4% [1, 3, 5]. In a review by Yeung et al., 3384 matched primary and metastatic pairs reported from 47 studies were analysed, ER expression median discordance between primary and metastatic site is 14% [12]. In respect to the general concordance, our result is consistence with the previous reports.

## Conclusion

ER expression pattern of primary breast tumor could be used to predict ER expression concordance between primary lesion and its matched synchronous metastatic lesions. High concordance of ER status between the primary lesions and the paired metastatic lymph nodes could be expected in patients with uniform ER expression in all the punched cores from primary lesions, no matter it is strong ER expression or negative at all. As discordance in ER status between primary breast cancer and metastatic lesion occurred in 13.0% of cases, ER status of the metastatic site should be assessed if possible, especially in patients with variant ER expression in primary sites.

## References

[1] Nedergaard L, Haerslev T, Jacobsen GK (1995). Immunohistochemical study of estrogen receptors in primary breast carcinomas and their lymph node metastases including comparison of two monoclonal antibodies. APMIS.

[2] Falck AK, Fernö M, Bendahl PO, Rydén L (2010). Does analysis of biomarkers in tumor cells in lymph node metastases give additional prognostic information in primary breast cancer. World J Surg.

[3] Ataseven B, Gologan D, Gunesch A (2012). HER2/neu, topoisomerase 2a, estrogen and progesterone receptors: discordance between primary breast Cancer and metastatic axillary lymph node in expression and amplification characteristics. Breast Care (Basel).

[4] Adamczyk A, Niemiec J, Ambicka A (2016). Survival of breast cancer patients according to changes in expression of selected markers between primary tumor and lymph node metastases. Biomark Med.

[5] Li MH, Hou CL, Wang C, Sun AJ (2016). HER-2, ER, PR status concordance in primary breast cancer and corresponding metastatic lesion in lymph node in Chinese women. Pathol Res Pract.

[6] Kuukasjärvi T, Karhu R, Tanner M (1997). Genetic heterogeneity and clonal evolution underlying development of asynchronous metastasis in human breast cancer. Cancer Res.

[7] Yang YF, Liao YY, Yang M, Peng NF, Xie SR, Xie YF (2014). Discordances in ER, PR and HER2 receptors between primary and recurrent/metastatic lesions and their impact on survival in breast cancer patients. Med Oncol.

[8] Sofi GN, Sofi JN, Nadeem R (2012). Estrogen receptor and progesterone receptor status in breast cancer in relation to age, histological grade, size of lesion and lymph node involvement. Asian Pac J Cancer Prev.

[9] Thompson AM, Jordan LB, Quinlan P (2010). Prospective comparison of switches in biomarker status between primary and recurrent breast cancer: the breast recurrence in tissues study (BRITS). Breast Cancer Res.

[10] Zhu X, Ying J, Wang F, Wang J, Yang H (2014). Estrogen receptor, progesterone receptor, and human epidermal growth factor receptor 2 status in invasive breast cancer: a 3,198 cases study at National Cancer Center, China. Breast Cancer Res Treat.

[11] Yi M, Huo L, Koenig KB (2014). Which threshold for ER positivity? A retrospective study based on 9639 patients. Ann Oncol.

[12] Yeung C, Hilton J, Clemons M (2016). Estrogen, progesterone, and HER2/neu receptor discordance between primary and metastatic breast tumours-a review. Cancer Metastasis Rev.

